# The potential value of microRNA-145 for predicting prognosis in patients with ovarian cancer: A protocol for systematic review and meta-analysis

**DOI:** 10.1097/MD.0000000000026922

**Published:** 2021-08-13

**Authors:** Zhen Chen, Zelan Xiao, Siheng Zeng, Zhiqiang Yan

**Affiliations:** Department of Gynecology, Hainan Western Central Hospital, Danzhou, Hainan Province, China.

**Keywords:** bioinformatics, meta-analysis, microRNA-145, ovarian cancer, prognosis, protocol

## Abstract

**Background:**

As an anticancer gene, microRNA-145 (miRNA-145) inhibits the growth, migration, and invasion of cancer cells, and inhibits tumorigenesis by targeting various genes that are abnormally expressed in tumors. However, whether miRNA-145 can be applied as a biomarker for potential prognosis of ovarian cancer still remains controversial. Therefore, this study further explored the prognostic value and mechanism of miRNA-145 in ovarian cancer through meta-analysis and bioinformatics analysis.

**Methods:**

Eligible studies were identified by searching the China National Knowledge Infrastructure, Chinese Biomedical literature Database, Chinese Scientific and Journal Database, Wan Fang database, PubMed, EMBASE, and Web of Science up to July 2021. Pooled hazard ratios with 95% confidence intervals for patient survival were calculated to investigate the effects of miRNA-145 on the prognosis of ovarian cancer. Survival curves of differential expression of miRNA-145 were analyzed by Oncomir. The target genes of miRNA-145 were predicted by miRTARbase and Diana-Tarbase V7.0 database. Enrichr database was applied to analyze the target genes by gene ontology and Kyoto Encyclopedia of Genes and Genomes pathways. Protein–protein interaction network of target genes was constructed from STRING database. Cytoscape software was used to screen the hub genes to meet the requirements. The Gene Expression Profiling Interactive Analysis database was applied to analyze the survival outcomes of hub genes.

**Results:**

The results of this meta-analysis would be submitted to peer-reviewed journals for publication.

**Conclusion:**

This study provides high-quality evidence to support the relationship between miRNA-145 expression and ovarian cancer prognosis. Through bioinformatics analysis, we further explored the mechanism of miRNA-145 in ovarian cancer and related pathways.

## 1 Introduction

Ovarian cancer is a common malignancy in women, with insidious onset, and it is difficult to be detected.^[1]^ Most diagnosed patients were at advanced stage. Clinically, the survival time of ovarian cancer patients is prolonged by surgery and postoperative adjuvant therapy, while the 5-year survival rate is still low.^[2,3]^ The exploration on tumor markers of ovarian cancer has important predictive value for optimizing treatment plan and prolonging survival time of patients.

MicroRNA (miRNA) is one kind of short noncoding RNAs, with only 20 to 24 nt in length, and involves in the posttranscriptional regulation of gene expression in multicellular organisms by affecting the stability and translation of mRNA.^[4]^ MiRNA is involved in many aspects of tumor regulation, including proliferation, apoptosis, invasion, metastasis, angiogenesis, drug resistance, and autophagy.^[5]^ As a noncoding single-stranded RNA small molecule, miR-145 is widely discovered in eukaryotic cells and is involved in the occurrence and development of tumor diseases.^[6–8]^ A number of studies have proved that miRNA-145 is abnormally expressed in a variety of tumors and is associated with clinicopathological stage and overall survival of tumor patients.^[9–11]^

Previous studies have revealed that miRNA-145 is considered a major tumor suppressor miRNA in ovarian cancer.^[12,13]^ Its expression is low in ovarian cancer tissues and cell lines, and its overexpression can inhibit the proliferation and migration of ovarian cancer cells.^[14]^ On the contrary, whether miRNA-145 can be used as a potential biomarker for ovarian cancer prognosis still remains controversial.^[14–16]^ To evaluate the association between miRNA-145 expression levels and survival outcomes in ovarian cancer patients, and to reduce differences and biases among reported studies, we performed a meta-analysis. In addition, this study applied bioinformatics to explore the association between miRNA-145 and the prognosis of ovarian cancer patients, and focused on the biological processes that miRNA-145 may be involved in ovarian cancer, so as to provide markers that are related to early diagnosis and prognosis of ovarian cancer.

## 2 Methods

### 2.1 Study registration

The protocol of the systematic review has been registered on Open Science Framework. The registration number is DOI 10.17605/OSF.IO/7GNHQ. This meta-analysis protocol is based on the Preferred Reporting Items for Systematic Reviews and Meta-analysis Protocols (PRISMA-P) Statement Guidelines.^[17]^

### 2.2 Data sources and search strategy

A literature search was conducted in the database of China National Knowledge Infrastructure, Chinese Biomedical literature Database, Chinese Scientific and Journal Database, Wan Fang database, PubMed, EMBASE, Web of Science, and the search time was up to July 2021. The search strategy for PubMed is shown in Table 1.

**Table 1:** Search strategy in PubMed database.

Number	Search terms
#1	Ovarian Neoplasms[MeSH]
#2	Cancer of Ovary[Title/Abstract]
#3	Ovarian Cancer[Title/Abstract]
#4	Cancer of the Ovary[Title/Abstract]
#5	Neoplasms, Ovarian[Title/Abstract]
#6	Ovary Cancer[Title/Abstract]
#7	Ovary Neoplasms[Title/Abstract]
#8	Cancer, Ovarian[Title/Abstract]
#9	Cancer, Ovary[Title/Abstract]
#10	Cancers, Ovarian[Title/Abstract]
#11	Cancers, Ovary[Title/Abstract]
#12	Neoplasm, Ovarian[Title/Abstract]
#13	Neoplasm, Ovary[Title/Abstract]
#14	Neoplasms, Ovary[Title/Abstract]
#15	Ovarian Cancers[Title/Abstract]
#16	Ovarian Neoplasm[Title/Abstract]
#17	Ovary Cancers[Title/Abstract]
#18	Ovary Neoplasm[Title/Abstract]
#19	or/1-18
#20	Hsa-miR-145[Title/Abstract]
#21	MicroRNA-145[Title/Abstract]
#22	miRNA-145[Title/Abstract]
#23	MiR-145[Title/Abstract]
#24	or/20-23
#25	Prognos^∗^[Title/Abstract]
#26	Survival [Title/Abstract]
#27	or/25-26
#28	#19 and #24 and #27

### 2.3 Inclusion criteria for study selection

Publications that studied patients with ovarian cancer; measured expression of miRNA-145 in human samples; and measured at least 1 survival curve of overall survival, disease-free survival, with or without hazard ratios (HRs) or 95% confidence intervals (CIs).

The exclusion criteria were review articles or letters; animal or cell experiments; and publications that lack key information such as HRs, 95% CIs, and *P* value.

### 2.4 Data collection and analysis

**Figure 1:**
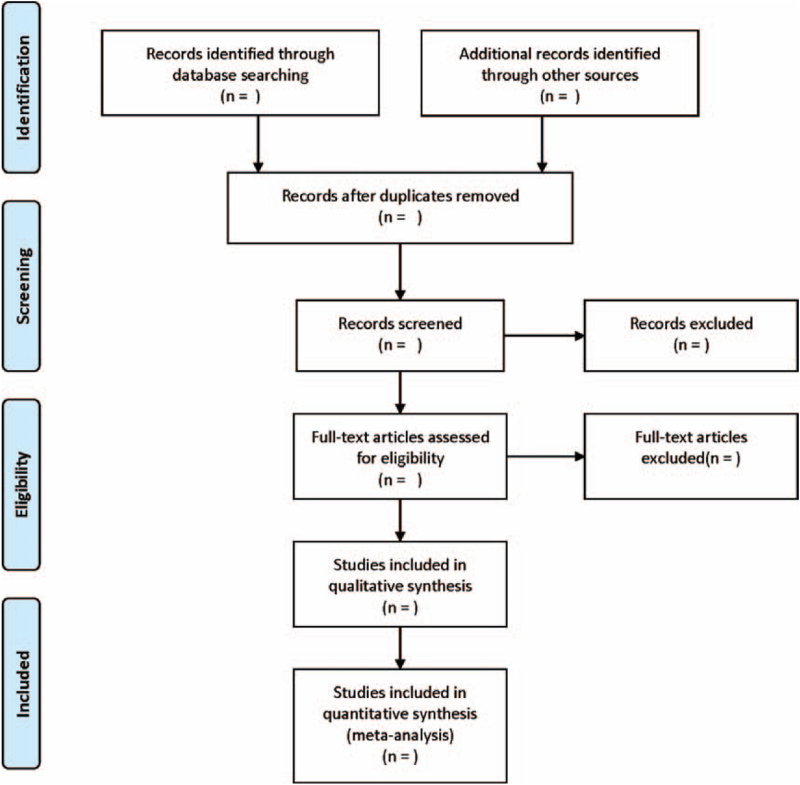
Flow diagram of study selection process.

The literature screening process is displayed in Figure 1. Two reviewers independently extracted data from eligible studies. The following information was extracted: first author, year of publication, country/region, ethnicity, number of cases, tumor stage, sample type, test method, follow-up and cutoff value, HRs, and the corresponding 95% CIs of miRNA-145 for overall survival and disease-free survival. If survival data were not directly available, the required data were obtained from the survival curve, with Engauge Digitizer 4.1 software being described by Tierney et al.^[18]^ Disagreements were resolved through comprehensive discussion and examined by a third investigator.

### 2.5 Assessment of quality in included studies

Literature quality was evaluated based on the Newcastle-Ottawa Quality Assessment Scale.^[19]^ The perfect score is 9 points, and Newcastle-Ottawa Quality Assessment Scale score ≥ 6 is classified as a high-quality study.^[20]^

### 2.6 Measures of prognosis

The HRs of the survival analysis were used, and the 95% CIs was applied to represent the effect level.

### 2.7 Management of missing data

If there exists insufficient or missing data in the literature, we would only analyze the currently available data and discuss its potential value.

### 2.8 Statistical analysis

STATA 16.0 (STATA Corporation, College Station, TX) was used for this meta-analysis. HRs and its 95% CIs were applied to evaluate the relationship between miRNA-145 expression and clinical prognosis in patients suffering from ovarian cancer. Heterogeneity among studies was tested by carrying out the Q test and *I*^*2*^ statistics. If *P* > .1 and *I*^*2*^ < 50%, the fixed-effects model was adopted. If not, the random-effects model was adopted.

### 2.9 Additional analysis

#### 2.9.1 Subgroup analysis

The subgroup analyses were conducted by adopting miRNA-145 testing methods, ethnicity, and survival data sources.

#### 2.9.2 Sensitivity analysis

Sensitivity analyses were performed by omitting each study at a time to assess the consistency and stability of the pooled results.

#### 2.9.3 Reporting bias

The publication bias was quantitatively analyzed adopting Egger linear regression method and the Begg rank correlation test.^[21,22]^

## 3 Bioinformatics analysis

### 3.1 Expression of miRNA-145 and prognosis of ovarian cancer

The survival curve of miRNA-145 differential expression was analyzed by OncomiR online database (http://www.oncomir.org/), and the correlation between miRNA-145 expression and prognosis of ovarian cancer patients was discovered.

### 3.2 Target gene prediction

The target genes of miRNA-145 were predicted by miTarbase (http://mirtarbase.cuhk.edu.cn/) and Dianatarbase V7.0 database (http://www.tarbase.com/),^[23]^ and the intersection target genes were obtained by Venn diagram intersection.

### 3.3 Gene ontology (GO) and Kyoto Encyclopedia of Genes and Genomes (KEGG) analysis

GO analysis and KEGG analysis were performed on the common target genes in Enrichir database (https://maayanlab.cloud/Enrichr/). On the other hand, we screened out *P* < .05 and the names of significantly enriched GO and KEGG pathway.

### 3.4 Protein–protein interaction network construction of target genes and HUB gene analysis

Protein–protein interaction network of target genes was constructed by String (https://www.string-db.org/), and its interaction data was imported into Cytoscape. Cytohubba plug-in and Degree method were adopted to screen out the top 20 target genes as hub genes, and the data results were visualized.

### 3.5 The relationship between Hub gene expression and prognosis of ovarian cancer

The survival curve of hub gene and ovarian cancer prognosis was plotted by Gene Expression Profiling Interactive Analysis database (http://gepia.cancer-pku.cn/).

### 3.6 Ethics

Our research data were derived from published literatures, because there existed no patient recruitment and personal information collection. Therefore, ethical approval was not required.

## 4 Discussion

Although the diagnosis and treatment of ovarian cancer have improved greatly in recent decades, the 5-year survival rate has not enhanced significantly.^[24,25]^ Therefore, it is of great clinical significance to search for biomarkers with high specificity and sensitivity for the prognosis of ovarian cancer.

MiRNA-145 is differentially expressed in many malignancies and is associated with tumorigenesis. More and more evidences indicated that miR-145 has antiproliferative and pro-apoptotic effects, and may be a miRNA with tumor suppressor effects.^[26]^ It was proved that miR-145 inhibited proliferation, invasion, and tumor growth of ovarian cancer cells and promoted apoptosis by negatively regulating the expression of target genes such as p53, high mobility group protein A2, C-Myc and p70S6K1.^[27,28]^ All of these findings suggested that miRNA-145 can serve as a novel prognostic marker and can be used as a therapeutic target for ovarian cancer.

We performed a meta-analysis to investigate the expression of miRNA-145 in ovarian cancer and its clinical prognostic significance. Meanwhile, bioinformatics method was adopted to analyze the expression of miRNA-145 in ovarian cancer and its potential biological process, so as to provide new ideas and therapeutic targets for further exploration on molecular mechanisms.

## Author contributions

**Conceptualization:** Zhiqiang Yan, Zhen Chen.

**Data curation:** Zelan Xiao.

**Formal analysis:** Zelan Xiao, Zhen Chen.

**Funding acquisition:** Zhiqiang Yan.

**Investigation:** Zelan Xiao.

**Methodology:** Zelan Xiao, Zhen Chen.

**Project administration:** Zhiqiang Yan.

**Resources:** Siheng Zeng.

**Supervision:** Zhiqiang Yan.

**Validation:** Siheng Zeng.

**Visualization and software:** Zhen Chen and Zelan Xiao.

**Visualization:** Siheng Zeng.

**Writing – original draft:** Zhen Chen and Zhiqiang Yan.

**Writing – review & editing:** Zhen Chen and Zhiqiang Yan.
